# In the eye of the beholder: Decision-making of lawyers in cases of sexual harassment

**DOI:** 10.1371/journal.pone.0272606

**Published:** 2022-08-11

**Authors:** Liza Zvi, Mally Shechory-Bitton

**Affiliations:** Department of Criminology, Ariel University, Ariel, Israel; University of Padova, ITALY

## Abstract

**Objective:**

The purpose of the present study was to examine the effect of deliberative vs. intuitive thinking styles on forensic judgments of legal professionals. Two hypotheses were tested: (a) that low deliberative thinking would be related to judgmental biases (b) that lawyers would report a greater tendency and preference toward deliberative thinking in comparison to students and make more rational judgments.

**Method:**

Ninety-one lawyers and 120 undergraduate students, who served as controls, were asked to read a criminal case depicting sexual harassment (SH) and judge victim and offender blame, whether the case constitutes SH, and the damage for the victim.

**Results:**

Deliberative thinking of lawyers was higher than students, and higher than their intuitive thinking style, supporting the more rational blame attributions of the former. In addition, higher deliberative thinking was related to a stronger perception of the event as SH. Nevertheless, all the participants were more inclined to perceive the case as SH when the victim was a woman instead of a man.

**Conclusions:**

The results suggest that gender stereotypes and bias may persist despite high deliberative thinking and may even be manifested through deliberative thinking processes. Awareness of legal professionals of these biases as well as the development of more objective tools which will help make the judging process less subjective—will ensure more accurate assessment of victims, offenders, and cases.

## Introduction

Making decisions in cases of sex offenses poses a difficult legal choice as these are often offenses where it is "word against word" (i.e. suspect versus victim [1]). Without clear evidence, legal professionals tasked with deciding a case may base their decisions on personal inferences, and bias might take place. This concern is reinforced by statistics showing that sexual assaults are characterized by high attrition rate, with attrition being the greatest at the investigative stage [2, 3].

Due to the fact that legal professionals are trained and experienced, it may be assumed that they make better decisions than the average person. The perception is that courts are based on logic and not on emotions and that the work of legal professionals has a rational basis [4]. For instance, the customary norms of legal practice teach lawyers to separate ethical considerations from their professional practice, to support their clients zealously, and to perform their job devoid of emotional involvement [5].

However, research findings show that legal professionals are often influenced by not only the facts in a case, but extralegal factors, and that social attitudes affect the legal and justice systems, infecting them with myths and stereotypes common in general society [6–11]. This was found to be especially true when evidence was lacking, allowing stereotypes and attitudes held by judges to register a pronounced impact [12]. Given these findings, considering the way in which legal professionals process information is essential and highly relevant to judicial biases. It is an even more important factor to consider in sexual assault cases, which are known to be highly emotionally inflammatory and contain many implicit stereotypes about victims and offenders [e.g., 13–15].

The research on legal professionals’ perceptions is mainly focused on police officer perceptions of rape incidents [e.g., 1, 10, 16], with findings indicating that police officers may hold rape myths and stereotypes and show biased perceptions of victims’ credibility and blame [e.g., 3, 16, 17]. Much less attention has been given to investigating perceptions and decision making processes of judges, as well as prosecutors, and to other sex offences other than rape. In a prosecutorial mode, lawyers make the decision to charge (or not) as well as determine the charges and charge reductions along with plea negotiations. They thus have a key role in the progression of cases through the criminal justice system [18–21].

The limited studies on prosecutorial discretion and decision making of judges show that, here too, decisions might be affected by extra-legal factors and subjected to bias [12, 22–24]. To the best of our knowledge and based on a comprehensive review of the literature, there are no studies on the relations between forensic judgments of lawyers and information processing style. Biased perceptions and decisions based on myths and stereotypes may indicate reliance on affect-related cognitions and suggest the use of heuristics. On the contrary, unbiased, evidence-based perceptions and decisions suggest a rational and logical information processing [25, 26]. Thus, the Cognitive Experiential Self Theory (CEST) [27, 28] seems to be an appropriate framework to test the relations between forensic decisions and judgments and rational or affect-related and heuristic thinking in the legal profession.

According to CEST, there are two different parallel pathways of information processing: the rational pathway and the experiential one. The first pathway occurs consciously, is effortful, analytical, logical and reason oriented. The second is preconscious, involves rapid automatic processing, using heuristics, and is highly affect-oriented. The degree to which individuals rely on either pathway is based on individual trait differences and situational demands [27–29]. Research has shown that the preferred decision-making method is using the rational system, and that the use of the intuitive mechanism might lead to bias in the way information is gathered and analyzed and in reaching a decision that fits the given situation [30]. Nevertheless, under certain conditions such as handling complex information, uncertainty, or time pressure, decisions made in an intuitive way using heuristics tailored to the decision environment can be as good and accurate as the rational deliberate ones [31–33].

Within the forensic field, CEST was evaluated mostly in relation to jury decision-making, showing that information processing style may be highly relevant to judicial biases [34–36]. It was generally shown that experiential processors are more susceptible to extralegal influences and may rely more on intuitive rather that evidence-based judgments [34], and that juries made better decisions when they were directed into a rational mode of information processing [37, 38].

In the present study, an attempt was made to broaden knowledge by examining this relation between forensic decisions and judgments and thinking styles among lawyers. It is of note that Israel, where the present study was undertaken, does not use the lay jury system. Instead, trials are presided by a judge or panel of judges. Thus, the way in which legal professionals process information is particularly relevant to judicial outcomes. However, considering the role of judges in overseeing and guiding trials in jury based criminal justice systems [e.g., 39–41], as well as the pivotal role of lawyers as prosecutors in criminal justice systems in general, such an inquiry is of relevance to various judicial systems.

In the present study, the decision making processes and judgments of lawyers will be analyzed with reference to a situation of sexual harassment (SH). The concept of SH is ambiguous, with no clear consensus on a universally accepted definition [see 42, 43 for a review]. Despite increased social awareness and legal definitions [44], deciding whether sexual harassment has occurred is deeply influenced by cultural schemas. For example, interpretation may depend on victim and offender gender [e.g., 45–47]. The present study is part of a research project aimed at assessing lawyers’ perceptions in cases of sexual harassment. A previous study within this project focused on assessing such effects and indicated discriminatory judgments of SH based on victim and offender gender [48].

SH may be regarded as both a civil wrong and a criminal offence. In Israel, SH is a violation of criminal law. The Sexual Harassment Prevention Act [49] states six situations which constitute SH: 1) extortion (using threats to coerce an individual into sexual activity); 2) indecent assault; 3) recurring sexual offers (multiple attempts by the harasser even after their original sexual proposition was rejected); 4) repeated references to a person focusing on sexuality (continuing even after the harasser was told to desist from said references); 5) disgraceful or degrading treatment of a person in relation to their sex or sexual orientation; and 6) posting a photograph, movie or recording of a person, focusing on their sexuality in circumstances in which the publication might humiliate or degrade the person, and without receiving consent for the publication. Looking at the wording of the Israeli law, there may be quite a few situations that may be open to interpretation, where the judicial system can find it difficult to decide whether or not SH occurred. This ambiguity reinforces the need for deeper knowledge and understanding as to how lawyers see these cases and process the information related to them.

The participants in the present study were lawyers working in law and in the courts. Student participants served as a control group. All the participants were asked to read a case depicting indecent assault, which constitutes a violation of section 3.A2 ("indecent assault") of the Israeli Sexual Harassment Prevention Act [49]. After reading, the participants were asked to report on their perceptions of blameworthiness of the offender and victim in various aspects. They were also asked to judge whether the depicted event constitutes SH and whether the behavior of the harasser caused the victim long-term mental damage.

Predictions were based on CEST theory and previous research testing the relationship between cognitive style and forensic judgments with lay participant samples. CEST maintains that the experiential system is the default mode of information-processing, because it is efficient and requires little effort. This may be true for lay people. Nevertheless, lawyers are taught and educated to rely on rational thinking and analysis of evidence. It was therefore hypothesized that lawyers would report a greater tendency and preference toward deliberative (i.e., rational) thinking in comparison to students. It was further hypothesized that resulting from their deliberative thinking, lawyers would make more rational judgments with respect to blame attributions and perceiving the scenario as SH. Bias was further assessed using a manipulation of the victim’s gender. There is ample research suggesting that female and male victims may receive differential treatment by lay people and legal professionals alike [e.g., 50–54]. Thus, we used two scenarios, identical in all aspects aside from victim and offender gender.

Finally, it was hypothesized that judgmental biases would be related to low deliberative thinking (i.e. low rationality). Deliberative thinking means weighing only relevant evidence and excluding any other irrelevant factors that might bias perception (e.g., victim/offender gender, personal attitudes and experiences, feelings, sentencing philosophy, etc.). Therefore, people that are high in deliberative thinking are expected to show less bias whereas people low in deliberative thinking are expected to exhibit much greater bias.

## Method

### Participants

Participants were 91 lawyers and 120 undergraduate students (a total of 211). Of the lawyers, 39 (42.9%) were women and 52 (57.1%) men. Of the students, 62 (51.7%) were men and 58 (48.3%) women. The participants were undifferentiated by sex (Z = 1.27, P>0.05). The mean age of the lawyers (M = 42.91, SD = 10.02) was significantly higher than that of the students (M = 24.12, SD = 2.51) (*t*(98.59) = 17.49, p < .001). All the students were undergraduates in the social sciences, at various schools. The lawyers had been working in their profession for a mean of 12.88 years (SD = 8.01).

### Instruments

#### Rational experiential inventory

To adequately assess individual use of rational (deliberative) and experiential (intuitive) processing modes, Epstein, Pacini, Denes-Raj and Heier [55] created the Rational Experiential Inventory (REI). Shortened versions of the REI are also in use [56], including a 24-item version [e.g., 33, 57], which was used in the present study. The REI and its shortened versions are considered valid and acceptable measures [e.g., 58–61]. The 24-item REI includes two unipolar scales, ranking participants on two dimensions of thinking style: rational analytic (e.g., I don’t think it is a good idea to rely on one’s intuition for important decisions; α = .85) and experiential-intuitive (e.g., I often go by my instincts when deciding on a course of action; = .86). Scores were composed of the mean of the items so that higher scores represent higher deliberative or intuitive cognitive styles (range 1–5). The correlation between deliberative and intuitive cognitive styles was *r* = -.03, *p* = .622.

#### Case scenarios

Two case scenarios depicted behavior that constitutes a violation of section 3.A2 ("indecent assault") of the Sexual Harassment Prevention Act [49]. The gender of the victim and of the offender was manipulated (either a male harasser and a female victim or a female harasser and a male victim). All other aspects were held constant. The following is a description of the male harasser / female victim scenario: "Sara was invited to a party at her friend’s house. One of the friends introduced Sara to David. The atmosphere was cheerful. Everyone danced, drank, and had a good time. Sara and David spent the evening together, talking flirtatiously, laughing, and touching each other. At a certain point David told Sara that he needed some rest. He went into the bedroom. Sara followed him and asked if everything is okay. He said that he has a headache and asked if she could make him a cup of tea. Sara was glad to oblige. When she returned to the room she found him lying on the bed naked. She gave him the cup of tea and turned to leave the room. He pulled her to him, hugged her, and kissed her on the neck and tried to insert his hand into her pants. Sara told him that she was tired and wanted to go home. She freed herself from his embrace, fled the room, and left the party in an agitated state".

#### Victim blame scale

Victim-blaming was measured using three items: To what extent—do you think that the victim can be blamed for the event? Do you think that the victim could have prevented the event? Do you think that the victim provoked the offender’s behavior? Cronbach’s α was .72.

#### Offender’s blame scale

Offender blame was measured using the three following items: To what extent—do you think that the offender can be blamed for the event? do you think that the victim should report to the police? Do you think that the offender should be prosecuted? Cronbach’s α was .78.

#### Judging the behavior as sexual harassment

The participants indicated the degree to which they perceived the event described in the scenario as sexual harassment (SH).

#### Damage caused to the victim

One item was used to indicate participant perceptions regarding long-term mental damage the victim may have suffered as a result of the SH: In your opinion, did the offender’s behavior caused the victim to suffer long term mental damage?

The questions the participants received were worded with the names of the victim and offender, depending on the given scenario.

Each of the above items was rated on a 5-point Likert-type scale ranging from 1 (*strongly disagree*) to 5 (*strongly agree*). Scores were comprised of item means, such that higher scores represent greater blame and greater belief that the behavior constitutes SH.

### Procedure

Data collection was goal-oriented, with only lawyers working in law and in the courts included in the study. Among the undergraduate students, data collection was done by research assistants who met with participants at their place of study. Participation in the study was on a voluntary basis. Participants signed an informed consent. They were told that stopping their participation in the study is possible at any point, with no penalty. Participants were also promised that the data would be used for research purposes only.

Participants were assigned at random to one of the two SH scenarios. Forty-six lawyers were presented with the woman as victim case and 45 with the man as victim. Sixty students were presented with the woman as victim case and 60 with the man as victim. No demographic differences were found within each group (lawyers/students) by victim gender in the scenario presented. The research was approved by the university’s institutional ethics committee.

### Data analysis

Data were analyzed with SPSS ver. 25. Internal consistencies were calculated, and variables were defined as item means. Variables of perception of the event were exponentially and log transformed due to non-normal distributions. Differences in the perception of the event and cognitive style, by group and the victim’s gender and their interaction were analyzed with 2X2 analyses of covariance, controlling for the respondent’s gender (1-males, 0-females). The respondent’s gender was controlled for, rather than serving as another independent variable, to avoid excessively small cells. The Bonferroni correction for multiple comparisons was applied in the analyses of variance.

Four multiple hierarchical regressions were calculated to assess the relationship between the perception of the event and cognitive style, group, victim’s gender, and their interactions. The regressions were calculated in four steps. The respondent’s gender was entered in the first step as a control variable. Cognitive style was entered in the second step as the independent variable. Group, victim’s gender, and their interaction were entered in the third step as the moderating variables. Finally, the two way and three way interactions between cognitive style and group and victim’s gender were stepwise entered in the fourth step. Continuous variables were standardized.

Post hoc power analysis has shown that for a 2X2 analysis of covariance, with an effect size of η^2^ = 0.04, α = 0.05, and N = 211, power level is 0.82. In cases where η^2^ < 0.04, power level is lower than 0.80 [62]. An additional post hoc power analysis has shown that for a regression with an effect size of η^2^ = 0.09 (the minimum found in this study), α = 0.05, N = 211, and 7 predictors, power level is 0.90. In all other regressions, power is 0.99 [62].

## Results

### Deliberative and intuitive cognitive thinking styles

Group differences in cognitive style were analyzed with a two way repeated measures analysis of covariance, controlling for the respondent’s gender (1-males, 0-females). The interaction between group and type of cognitive style (deliberative vs. intuitive) was analyzed with repeated measures, in order to assess the difference between the two styles within group (Table 1).

**Table 1:** Means, standard deviations, and F values for cognitive style, by group (N = 211).

	Lawyers (*N* = 91)		Students (*N* = 120)		Difference		
	Deliberative *M* (*SD*)	Intuitive *M* (*SD*)	Deliberative *M* (*SD*)	Intuitive *M* (*SD*)	Group *F*(1, 208) (η^2^)	Cognitive style *F*(1, 208) (η^2^)	Group x Cognitive style *F*(1, 208) (η^2^)
Cognitive style	3.85 (0.64)	3.64 (0.65)	3.65 (0.72)	3.72 (0.66)	0.55 (.003)	4.43* (.021)	6.60* (.031)

*p < .05

M–mean, SD–standard deviation, η^2^ –effect size.

Results show that the interaction between group and cognitive style is significant, yet with a small effect size. Its interpretation revealed that the deliberative cognitive style was higher among lawyers than students (*F*(1, 208) = 5.04, *p* = .026, η^2^ = .024), and no group differences were found for the intuitive cognitive style (*F*(1, 208) = 1.79, *p* = .182, η^2^ = .009). Further, the deliberative cognitive style was higher than the intuitive cognitive style among lawyers (*F*(1, 208) = 6.03, *p* = .015, η^2^ = .028), but no differences were found among students (*F*(1, 208) = 1.21, *p* = .273, η^2^ = .006).

### Blame attributions, perception of behavior and consequences

Differences between groups in blame attributions, in perception of the event and consequences for the victim, by group and victim’s gender (2X2), were analyzed with two way analyses of covariance, controlling for the respondent’s gender (1-males, 0-females).

As can be seen in Table 2, attribution of Offender’s blame was found to be higher among lawyers than among students (*F*(1, 206) = 4.44, *p* = .036, η^2^ = .021) with a low effect size, and higher for female victims than male victims (*F*(1, 206) = 23.77, *p* < .001, η^2^ = .104) with a moderate effect size. The interaction between group and the victim’s gender was significant (*F*(1, 206) = 8.61, *p* = .004, η^2^ = .040) with a low effect size, such that the attribution of blame to female offenders by students (i.e., male victims) was lower than all other sub-groups. Attribution of victim blame was higher among students than among lawyers (*F*(1, 206) = 7.11, *p* = .008, η^2^ = .033) with a low effect size. No other differences were found.

**Table 2:** Means, standard deviations, and F values for blame attributions and perception of the event by group and victim’s gender (N = 211).

Group	Lawyers (*N* = 91)			Students (*N* = 120)			Total (*N* = 211)			Difference		
Victim’s gender:	Male *M* (*SD*)(*n* = 45)	Female *M* (*SD*) (*n* = 46)	Total *M* (*SD*)	Male *M* (*SD*) (*n* = 60)	Female *M* (*SD*) (*n* = 60)	Total *M* (*SD*)	Male *M* (*SD*)	Female *M* (*SD*)	Total *M* (*SD*)	Group *F*(1, 206) (η^2^)	Victim’s gender *F*(1, 206) (η^2^)	Group x victim’s gender *F*(1, 206) (η^2^)
Offender’s blame	3.86 (0.96)	4.05 (0.98)	3.96 (0.97)	3.13 (0.93)	4.17 (0.83)	3.65 (1.02)	3.45 (1.01)	4.12 (0.90)	3.78 (1.01)	4.44* (.021)	23.77*** (.104)	8.61** (.040)
Victim’s blame	1.63 (0.66)	1.83 (1.03)	1.73 (0.87)	2.11 (0.85)	1.91 (0.80)	2.01 (0.83)	1.90 (0.81)	1.87 (0.90)	1.89 (0.86)	7.11** (.033)	0.09 (.001)	2.01 (.010)
Damage caused to the victim	2.78 (0.97)	3.14 (1.15)	2.96 (1.08)	2.58 (1.15)	3.87 (0.89)	3.23 (1.21)	2.67 (1.07)	3.56 (1.07)	3.11 (1.16)	4.23* (.020)	31.89*** (.135)	9.97** (.047)
Behavior is sexual harassment	4.29 (0.84)	4.86 (0.51)	4.57 (0.75)	3.77 (1.25)	4.73 (0.66)	4.24 (1.11)	3.99 (1.12)	4.79 (0.60)	4.38 (0.99)	4.82* (.023)	51.51*** (.202)	0.39 (.002)

*p < .05,

**p < .01,

***p < .001

M–mean, SD–standard deviation, η^2^ –effect size.

Assessment of the mental damage caused to the victim was higher among students than among lawyers (*F*(1, 206) = 4.23, *p* = .041, η^2^ = .020) with a low effect size, and regarding female victims than male victims (*F*(1, 206) = 31.89, *p* < .001, η^2^ = .135) with a moderate effect size. The interaction between group and the victim’s gender was significant (*F*(1, 206) = 9.97, *p* = .002, η^2^ = .047) with a low effect size, so that the attribution of damages to female victims by students was higher than all other sub-groups.

The behavior was regarded as sexual harassment more highly by lawyers than by students (*F*(1, 206) = 4.82, *p* = .029, η^2^ = .023) with a low effect size, and more highly regarding female victims than male victims (*F*(1, 206) = 51.51, *p* < .001, η^2^ = .202) with a large effect size. The interaction between group and the victim’s gender was non-significant.

### Relations between the research variables

Four multiple linear regressions (OLS) were calculated to assess the relationship between the perception of the event and cognitive style, group, victim’s gender, and their interactions. The regressions were calculated in four steps. The respondent’s gender was force entered in the first step as a control variable. Cognitive style was force entered in the second step as the independent variable. Group, victim’s gender, and their interaction were force entered in the third step as the moderating variables. Finally, the two way and three way interactions between cognitive style and group and victim’s gender were stepwise entered in the fourth step. As can be seen in Table 3, the four regression models were found significant, explaining 9% to 26% of the variance in the perception of the event.

**Table 3:** Multiple linear regressions for the perception of the event by group, victim’s gender, and cognitive style (*N* = 211).

	Offender’s blame		Victim’s blame		Damage to the victim		Behavior is sexual harassment	
Step 1	*B* (*SE*)	95%*CI*	*B* (*SE*)	95%*CI*	*B* (*SE*)	95%*CI*	*B* (*SE*)	95%*CI*
Respondent’s gender	-0.46 (6.92)	-14.10, 13.19	0.13 (0.06)*	0.02, 0.25	-0.37 (0.16)*	-0.68, -0.06	-14.81 (7.65)	-29.90, 0.27
Adj. *R*^2^	.001		.019		.021*		.013	
Step 2								
Respondent’s gender	0.36 (7.19)	-13.83, 14.54	0.13 (0.06)*	0.02, 0.25	-0.23 (0.17)	-0.56, 0.09	-16.29 (7.99)*	-32.05, -0.54
Deliberative cognitive style	7.22 (3.45)*	0.42, 14.02	-0.10 (0.03)***	-0.16, -0.05	-0.13 (0.08)	-0.28, 0.03	10.55 (3.94)**	2.77, 18.33
Intuitive cognitive style	3.91 (3.58)	-3.15, 10.97	-0.03 (0.03)	-0.09, 0.03	0.18 (0.08)*	0.02, 0.35	2.03 (3.98)	-5.82, 9.89
Adj. *R*^2^	.012		.072***		.045**		.039*	
Step 3								
Respondent’s gender	3.38 (6.67)	-9.77, 16.53	0.11 (0.06)	0.00, 0.23	-0.22 (0.15)	-0.52, 0.07	-13.42 (7.23)	-27.67, 0.83
Deliberative cognitive style	5.41 (3.22)	-0.94, 11.77	-0.09 (0.03)**	-0.15, -0.03	-0.13 (0.07)	-0.27, 0.01	9.70 (3.58)**	2.63, 16.76
Intuitive cognitive style	5.19 (3.32)	-1.36, 11.74	-0.04 (0.03)	-0.10, 0.02	0.18 (0.08)*	0.03, 0.34	2.54 (3.60)	-4.57, 9.65
Group	-6.10 (8.98)	-23.81, 11.60	-0.06 (0.08)	-0.22, 0.10	-0.63 (0.20)**	-1.02, -0.23	2.98 (9.62)	-15.99, 21.95
Victim’s gender	-49.97 (8.40)***	-66.54, -33.40	0.09 (0.07)	-0.05, 0.24	-1.31 (0.19)***	-1.68, -0.94	-54.34 (8.99)***	-72.06, -36.61
Group X victim’s gender	37.73 (12.84)**	12.41, 63.04	-0.15 (0.11)	-0.38, 0.07	0.94 (0.29)**	0.38, 1.51	16.69 (13.76)	-10.45, 43.82
Adj. *R*^2^	.165***		.091***		.234***		.226***	
Step 4								
Respondent’s gender	3.40 (6.57)	-9.56, 16.36	--	--	-0.23 (0.15)	-0.52, 0.06	--	--
Deliberative cognitive style	13.37 (4.35)**	4.79, 21.94	--	--	0.07 (0.10)	-0.13, 0.26	--	--
Intuitive cognitive style	5.17 (3.27)	-1.28, 11.62	--	--	0.18 (0.08)*	0.03, 0.33	--	--
Group	-7.07 (8.85)	-24.52, 10.39	--	--	-0.65 (0.20)**	-1.04, -0.26	--	--
Victim’s gender	-52.27 (8.32)***	-68.68, -35.86	--	--	-1.37 (0.18)***	-1.73, -1.00	--	--
Group X victim’s gender	42.92 (12.80)***	17.68, 68.15	--	--	1.06 (0.28)***	0.50, 1.62	--	--
Deliberative cognitive style X victim’s gender	-16.90 (6.32)**	-29.36, -4.44	--	--	-0.40 (0.14)**	-0.68, -0.13	--	--
Total Adj. *R*^2^	.189***		.091***		.260***		.226***	
*F*	*F*(7, 203) = 7.96***		*F*(6, 204) = 4.50***		*F*(7, 203) = 11.45***		*F*(6, 204) = 11.13***	

*p < .05,

**p < .01,

***p < .001

‘Offender’s blame’ and ‘Behavior is sexual harassment’ were exponentially transformed,

‘Victim’s blame’ was log transformed, and ‘Damage to the victim’ did not deviate from normal distribution.

*B*–unstandardized coefficient, *SE*–standard error of B, 95%*CI–* 95% confidence interval of B.

Offender’s blame: 19% of the variance in the attribution of blame to the offender was explained in the model. A positive relationship was found between deliberative cognitive style and attribution of blame to the offender, so that higher deliberative thinking style was related to greater attribution of blame to the offender. The interaction between deliberative thinking and the victim’s gender was significant as well (β = -.24, *p* = .008). Its interpretation with simple slopes [63, 64] revealed a positive relationship between deliberative thinking style and attribution of blame to the offender regarding female victims (coefficient = 13.36, *SE* = 4.35, *t* = 3.07, *p* = .002, 95%*CI* = 4.79, 21.94), and no significant relationship regarding male victims (coefficient = -3.54, *SE* = 4.61, *t* = -0.77, *p* = .444, 95%*CI* = -12.63, 5.56). That is, higher deliberative thinking style was related with a greater attribution of blame to the offender regarding female, but not male, victims. In addition, the victim’s gender and the interaction between group and the victim’s gender were significant, as detailed in Table 2.

Victim’s blame: 9% of the variance in the attribution of blame to the victim were explained in the model. A negative relationship was found between deliberative thinking style and attribution of blame to the victim, so that a higher deliberative thinking style was related to a lower attribution of blame to the victim. All interactions were not significant.

Damage caused to the victim: 26% of the variance in the severity of the damage attributed to the victim was explained in the model. A positive relationship was found between intuitive thinking style and the severity of the consequences attributed to the victim, so that a higher intuitive thinking style was related to the attribution of more severe damage to the victim. In addition, the interaction between deliberative thinking style and the victim’s gender was significant (β = -.24, *p* = .005). Its interpretation with simple slopes (Aiken & West, 1991; Dawson, 2014) revealed a negative relationship between deliberative thinking style and attribution of severe consequences for the victim regarding male victims (coefficient = -0.34, *SE* = 0.10, *t* = -3.31, *p* = .001, 95%*CI* = -0.54, -0.14), and no significant relationship regarding female victims (coefficient = 0.07, *SE* = 0.10, *t* = 0.67, *p* = .503, 95%*CI* = -0.13, 0.26). That is, higher deliberative thinking style was related to the attribution of less severe consequences regarding male, but not female, victims. Furthermore, group, the victim’s gender and the interaction between group and the victim’s gender were significant, as detailed in Table 2.

Perception of the behavior as sexual harassment: 23% of the variance in the perception of the behavior as sexual harassment was explained in the model. A positive relationship was found between deliberative thinking style and the perception of the behavior as sexual harassment, so that a higher deliberative thinking style was related to a stronger perception of the behavior as sexual harassment. In addition, the victim’s gender was significant, as detailed in Table 2, and all interactions were not significant.

## Discussion

The purpose of the present study was to examine lawyers’ forensic decision making and judgments of victims and offenders in a situation involving SH in comparison to undergraduate students. As expected, deliberative cognitive style was higher among lawyers than students. Moreover, no group differences were found for the intuitive cognitive style. Deliberative cognitive style was higher than the intuitive cognitive style among lawyers, but no differences between the styles were found among students.

According to Ayal, Zakay, & Hochman [57], deliberative but not intuitive thinking is the crucial predictor of rational behavior. Initial intuitive preferences may or may not be manifested depending on the strength of the deliberative process. Thus, among individuals characterized by high deliberative thinking, the deliberative thinking process adjusts or overrides the initial intuitive preferences, directing behavior towards a rational solution. The significant gap in the magnitude of the two thinking styles among lawyers in the present study indicates that lawyers have a better capability to manage intuition and reach a correct forensic decision. This is supported by lawyers’ more rational blame attributions and perception of the event. It is possible that the process of their professional training has led lawyers to adopt a more rational, analytical and directed way of thinking, as this is essential to their work. On the other hand, it is also possible that more rational and analytical people are attracted to advocacy, because this is an area where these tendencies can be expressed.

Nevertheless, notwithstanding lawyers’ rational thinking, and although they were significantly less victim-blaming than students, analysis of the responses as a function of the victim’s gender revealed stereotypical thinking and gender-related bias. The Sexual Harassment Prevention Act [49] does not distinguish between female and male victims, and it was therefore expected that lawyers’ perceptions would reflect this position, especially in light of their hypothesized tendency for deliberative thinking. However, while their tendency toward deliberative thinking was indeed evident, similar to students, lawyers were more inclined to perceive the case as SH when the victim was a woman.

These findings correspond with previous findings indicating differential perception of men and women as victims and sexual assailants, showing that both men and women are less tolerant of sexual harassment when the victim is female than when the victim is male [65, 66]. Nevertheless, the research on social perceptions toward male victims of sex offences is relatively limited, and some studies have shown that perception was related to the seriousness of harassment, such that the more serious the behavior, the more it was perceived as SH, unrelated to victim or offender gender [67]. Recognizing that perceptions of victims might be influenced by gender stereotypes, the findings may reflect stereotypical perceptions related to the gender roles of men and women in society. Specifically, that men are more easily seen as offenders, whereas women are more easily seen as victims. According to the sociocultural model proposed in the 1980s by Tangri and her colleagues [68], men are stereotypically perceived as strong and resilient whereas women are perceived as weak. In the process of socialization, males are taught and rewarded for aggressive and dominating behavior and women are taught and rewarded for passive and avoidant behavior [68]. A woman sexually harassing a man contradicts these gender role expectations, which may be hard to reconcile. This may result in minimization of women’s aggressive behavior, or the damages caused to male victims, as the present findings show.

According to the chivalry or paternalism argument, gender stereotypes (e.g., females are delicate and naïve wives, daughters, and mothers) may lead to preferential treatment of female offenders [69]. Empirical research supports this notion by showing that female offenders receive more lenient treatment than similarly situated male offenders [69, 70] and have consistently lower incarceration odds than males convicted of similar offenses [70–74]. Legal professionals may also be biased. A recent study with a police officer sample showed that both officers and the control group of students were more inclined to send a male swindler to prison than a female swindler, whom they preferred to sentence to probation or rehabilitation [52]. The results may indicate such a chivalry bias among lawyers with respect to SH.

Overall, the findings suggest that instilled social gender-related perceptions may have overpowered lawyers’ tendency toward deliberative rational thinking. This conclusion is reinforced by the fact that deliberative thinking was found to be related to blaming the male offender, but not the female offender, as well as by the relationship between high deliberative thinking and minimization of the consequences to the male victim, but not to the female victim. Another possibility is that gender related bias took place via deliberative processes. According to System Justification Theory (SJT) [75], "people are motivated to justify and rationalize the way things are, so that existing social, economic, and political arrangements tend to be perceived as fair and legitimate" [76, p. 260]. If a woman sexually harassing a man is a threat to the traditional sexist conceptions of gender roles and behavior, it may trigger attempts to justify and rationalize the status quo (for example, via minimization of female aggressiveness and male victimization). Such justifications and rationalizations may take place via deliberative effortful thinking.

General behavior research has shown that individuals with a high deliberative thinking style are less prone to judgmental biases and make more rational choices [33, 57]. Similarly, within the forensic field, it was shown that juries made better decisions when they were directed into a deliberative mode of information processing [37, 38]. The present findings suggest that biases may be related not only to low deliberative thinking but may also be manifested via processes of high deliberative thinking. Specifically, in situations where judges are motivated to protect existing inner perceptions.

The predictions of the present study were with respect to low or high deliberative thinking. Yet, the findings indicated an interesting relation between a high intuitive thinking style and attribution of more severe mental damage to the victim. This may be explained by intuitive thinkers’ susceptibility to the influence of extralegal factors and emotions, as such an assessment is purely subjective. In a study by Lieberman [35], mock jurors primed to process information experientially were biased by an extralegal factor (attractiveness of the defendant) in awarding damages to the plaintiff, whereas the extralegal factor had no effect in the rational processing group. Another study [77] found that the more experiential the mock jurors’ processing state, the more likely they were to be influenced by “feelings about the case”. The present results with a sample of lawyers and sexual harassment scenario add to the existing evidence on the susceptibility of the intuitive system to the effect of emotions and extralegal factors. Practical implications are that intuitive thinkers, both legal practitioners and lay juries, may be more prone to assess psychiatric injuries and award victims with monetary damages.

Several limitations of the present study should be addressed. First, the present scenario did not include additional information on the offender or victim aside from their gender. Real life cases are characterized by many additional details (e.g., victim and offender age, physical appearance, social-economic status, race, etc.). It would be interesting to look at the effect of various victim and offender characteristics on judgments with respect to the two thinking styles. Another limitation is the relatively small sample size, as locating lawyers was an obstacle. It was therefore not possible to examine gender differences between the respondents, although all the statistical analyses included controlling for the respondent’s gender. Other participant characteristics like age should be examined, as there are some indications that perceptions of sexual harassment may vary with age [78]. It is also advised to test for control groups from the general population. Although rape research has found that observers from the general population make similar judgments of blame as do student participants, students represent an educated group associated with more liberal attitudes and a lower tendency to accept stereotypes [79]. Future research should examine the topic in larger and more diverse populations, manipulating various extralegal characteristics.

Participants in the present study were both civil and criminal lawyers. Differences related to specific legal area within the general occupation of lawyers may be examined as well.

While judgmental biases cannot be completely neutralized, it can be assumed that early awareness of these biases will serve as a reminder and help professionals focus on relevant considerations when it comes to evaluating others. The importance of the present research is in raising awareness of the existence of cognitive biases and, hence, to the importance of trying to minimize their impact when it comes to deciding fates. Development of more objective tools, which will help make the judging process less subjective, will ensure more accurate assessment of victims, offenders and cases. Greater reliability in judgments and decision-making processes is of great importance in criminal law, as well as in education, academia, the labor market and many other aspects of life.

## Supporting information

S1 File Dataset of the participants responses. (XLSX)
